# C-reactive protein polygenic risk is associated with obesity-related traits in schizophrenia spectrum disorders

**DOI:** 10.3389/fpsyt.2026.1873942

**Published:** 2026-07-14

**Authors:** Chenxu Zhao, Elnaz Naderi, Tesfa Dejenie Habtewold, Therese van Amelsvoort, Wiepke Cahn, Lieuwe de Haan, Marieke van der Pluijm, Claudia J.P. Simons, Jim van Os, Wim Veling, Richard Bruggeman, Behrooz Z. Alizadeh

**Affiliations:** 1 Department of Epidemiology, University Medical Center Groningen, University of Groningen, Groningen, Netherlands; 2 Department of Psychiatry and Neuropsychology, Maastricht University Medical Center, School for Mental Health and Neuroscience, Maastricht, Netherlands; 3 Brain Centre Rudolf Magnus, Department of Psychiatry, University Medical Center Utrecht, Utrecht University, Utrecht, Netherlands; 4 Altrecht, General Mental Health Care, Utrecht, Netherlands; 5 Department of Psychiatry, Amsterdam UMC, University of Amsterdam, Amsterdam, Netherlands; 6 Arkin Institute for Mental Health, Amsterdam, Netherlands; 7 GGzE Institute for Mental Health Care, Eindhoven, Netherlands; 8 Department of Psychosis Studies, Institute of Psychiatry, King’s College London, King’s Health Partners, London, United Kingdom; 9 Department of Psychiatry, University Medical Center Groningen, University of Groningen, Groningen, Netherlands

**Keywords:** cardiometabolic outcome, C-reactive protein, interleukin 6, obesity-related traits, polygenic risk score, schizophrenia spectrum disorders

## Abstract

**Background:**

Patients with schizophrenia spectrum disorders (SSDs) are at increased risk of cardiometabolic dysregulations, which substantially contribute to cardiovascular morbidity and reduced life expectancy. Chronic low-grade inflammation is a key factor in the development of cardiometabolic outcomes. Polygenic risk scores (PRS) for inflammatory biomarkers like for C-reactive protein (CRP) and interleukin 6 (IL-6) may help clarify the genetic contribution to this risk, yet evidence in SSDs populations remains limited.

**Methods:**

We investigated the associations of PRSes for CRP and IL6 with cardiometabolic outcomes in 671 patients with SSDs from the longitudinal Dutch Genetic Risk and Outcome in Psychosis (GROUP) study. Seven PRS_CRP_ and seven PRS_IL-6_ were constructed using clumping and threshold method at seven *P*-value thresholds (*P*_t_x_) based on large, independent genome-wide association studies. We examined 11 cardiometabolic outcomes measured at three years after diagnosis, including body mass index (BMI), waist circumference (WC), lipid levels, blood pressures, glycaemic markers, and a metabolic composite score. Linear regression models adjusted for age, sex, and population substructure tested the associations between PRSes, with multiple testing correction and bootstrapping for validation. Regression coefficients (β*_P-threshold_*) and 95% confidence intervals and unadjusted R^2^ (variance explained) were reported. Sensitivity analyses were conducted by further adjusting for smoking and antipsychotic medication use.

**Results:**

Higher standardized PRS_CRP_ was significantly associated with increased BMI (β_Pt_0.5_ = 0.64, 95%CI=0.21-1.02, *P_bootstapping_* = 0.003) and WC (β_Pt_0.5_ = 2.25, 1.00-3.53, *P_bootstapping_* < 0.001), explaining up to 1.85% variance in BMI, and 2.52% in WC. Nominal associations were also observed between PRS_CRP_ and triglycerides (TG) levels (β_Pt_0.2_ = 0.13, 0.01-0.26, *P_bootstapping_* = 0.036), and metabolic composite score (β_Pt_0.2_ = 0.14, 0.04-0.24, *P_bootstapping_* = 0.006), and between PRS_IL-6_ and HbA1c level (β_Pt_5e06_=-0.66, -1.26 to -0.05, *P_bootstapping_* = 0.033); however these associations did not remain significant after correction for multiple testing.

**Conclusions:**

Higher genetic susceptibility for low grade inflammation as captured by PRS_CRP_ is modestly but robustly associated with increased levels of obesity-related traits in SSDs independent from antipsychotics use. These results support CRP-related genetics pathways as potential contributors to risk of cardiometabolic vulnerability in SSDs and may inform genetic-based personalized risk stratification and prevention strategies in SSDs patients.

## 1 Introduction

Metabolic syndrome (MetS) is a cluster of metabolic dysregulations that heighten the risk of developing cardiovascular diseases, including insulin resistance, type II diabetes, dyslipidemia, central obesity, and high blood pressure (1, 2). Its prevalence is notably higher among individuals with schizophrenia spectrum disorders (SSDs) compared to the general population (3–5). Our recent research indicates that MetS and its components are linked to cognitive impairments in SSDs patients (6). Additionally, MetS significantly impacts quality of life and reduces life expectancy in this group. Addressing MetS in SSDs patients is crucial for improving health outcomes and overall well-being (7–10).

Classical inflammation involves a series of physiological immune responses to injury, marked by redness (rubor), swelling (tumor), heat (calor), and pain (dolor) (11). However, chronic low-grade inflammation is a risk factor for both somatic (12–15) and psychiatric (16) disorders and a primary suspect associated with cardiometabolic outcomes in patients with SSDs (17). C-reactive protein (CRP) is a commonly used sensitive biomarker for measuring chronic inflammation (18). Studies have shown elevated circulating levels of CRP in patients with SSDs (19) and cardiometabolic dysregulations (20, 21). Additionally, pro-inflammatory cytokines, especially interleukin-6 (IL-6), are often increased in these patients (22, 23). Elevated levels of CRP and IL-6 are associated with cardiometabolic outcomes like MetS, Type 2 Diabetes Mellitus (T2DM), and dyslipidemia (24–27) both in the general population and among those with SSDs. These findings highlight the significant connection between inflammation and cardiometabolic health outcomes.

Single nucleotide variants (SNVs) can slightly increase the risk of heritable diseases or traits (28, 29). The polygenic risk score (PRS) offers a more comprehensive tool for predicting disease susceptibility by evaluating the combined effect of multiple SNVs. PRS is calculated by a weighted sum of risk alleles in an individual’s genome (30). Given their established roles in low-grade systemic inflammation, their complementary positions within the inflammatory cascade, and the availability of large-scale genome-wide association studies (GWAS) summary statistics, CRP and IL-6 were selected as the inflammatory biomarkers of interest in the present study. Serum CRP and IL-6 levels are polygenic traits with a moderate heritability of 0.10 to 0.65 (31, 32), and 0.15 to 0.61 (33–35), respectively. Our prior research through meta GWAS has identified 293 independent SNVs that collectively explain up to 16.3% of the variance in CRP levels in general population (36, 37). Additionally, 94 SNVs at two independent genetic loci were significantly associated with IL-6 levels at a genome-wide level (at *P* < 5x10^−8^) (38). These findings indicate the potential of the PRS for CRP (PRS_CRP_) and IL-6 (PRS_IL-6_) as research and clinically relevant tools for predicting risk related to low-grade inflammation.

Inflammation and cardiometabolic outcomes are highly genetically correlated. Previous research (39) showed an overlap between inflammation and cardiometabolic outcomes for 13 genetic regions. Fotios et al (40) found a wide pleiotropic effect on cardiometabolic pathways of 19 gene sets that are associated with CRP levels. Likewise, another study found pleiotropic genetic correlations among eight cardiometabolic outcomes and nine inflammatory biomarkers including CRP and IL-6 (41). A recent study showed that PRS_CRP_ was associated with incident coronary heart disease risk in the general population (42). Nonetheless, the relationship between inflammatory-associated SNVs and their corresponding PRSes and cardiometabolic outcomes in patients with SSDs remains insufficiently studied.

We investigated the association between PRS_CRP_ and PRS_IL-6_ and cardiometabolic outcomes in a large longitudinal multi-center Dutch cohort of patients with SSDs. We hypothesized that PRS_CRP_ and PRS_IL-6_ are associated with cardiometabolic outcomes in this population.

## 2 Methods

### 2.1 Study design and participants

Data were extracted from the Genetic Risk and Outcome of Psychoses (GROUP) cohort study, a multi-center longitudinal investigation with a total of 3, 683 individuals, including 1, 119 patients with SSDs, 1, 059 siblings, 919 parents, and 586 healthy controls. The current study only included patients. Patients were enrolled in the GROUP study based on the following criteria: i) age range of 16 to 50 years at baseline, encompassing both extremes; ii) a diagnosis of non‐affective psychotic disorder according to the Diagnostic and Statistical Manual of Mental Disorders, Fourth Edition (DSM‐IV) criteria (43); iii) proficiency in the Dutch language; and iv) the ability and willingness to provide written informed consent. Data collection occurred at enrollment, with subsequent follow-up measurements conducted at 3 years and 6 years after enrollment. For the current analysis, data (GROUP official release number 8) from the 3-year follow-up was used given that cardiometabolic data were only collected at this time point from all participating institutes. The study protocol was centrally approved by the Ethical Review Board of the University Medical Center Utrecht and by local review boards of each participating institute. Details regarding sample characteristics, recruitment and assessment procedures are documented elsewhere (44). This structured approach ensures comprehensive data collection and adherence to ethical standards, facilitating reliable longitudinal analysis of the cohort.

### 2.2 Measurements

During the 3-year follow-up, participants underwent a comprehensive physical examination and blood tests to gather data on body mass index (BMI; kg/m^2^), waist circumference (WC; cm), pulse rate (PR; beats/min), systolic (SBP; mmHg) and diastolic blood pressure (DBP; mmHg). Mean arterial pressure (MAP; mmHg) was estimated following the formula: MAP = DBP + 1/3 (SBP-DBP). The glucose and lipid biomarkers including glycated hemoglobin (HbA1c; mmol/mol), low density lipoprotein (LDL; mmol/l), high density lipoprotein (HDL; mmol/l) cholesterol and triglycerides (TG; mmol/l) levels were measured in whole blood samples.

MetS was defined according to the US National Cholesterol Education Programme Adult Treatment Panel III (NCEP-ATP-III) criteria, where the presence of any three of the following five features constituted a diagnosis of MetS (45): i) fasting plasma glucose ≥100 mg/dl (equivalent to 5.6 mmol/l) or the use of antidiabetic medications (46). In cases where plasma glucose levels were not available, a HbA1c ≥5.1% (equivalent to 32 mmol/mol) was used as a criterion (47); ii) HDL cholesterol <50mg/dl (equivalent to 1.30 mmol/l) in woman or <40 mg/dl (equivalent to 1.03 mmol/l) in men; or the use of HDL increasing drugs; iii) TG ≥150 mg/dl (equivalent to 1.7 mmol/l) or the use of triglyceride-lowering drugs; iv) BP ≥130/85 mmHg or the use of antihypertensives; v) WC ≥88 cm in women or 102 cm in men. Standardized values of MAP, HDL, TG, WC, and HbA1c were calculated using patients’ means and standard deviations (SD) ranging within healthy reference values (48). Metabolic composite score (MCS), derived from the core MetS components (HbA1c, HDL, TG, blood pressure, and WC), was calculated by dividing the sum of all standardized components by five (48) and treated as a continuous measure of overall metabolic dysfunction.

Cardiometabolic outcomes included the above 10 individual cardiometabolic traits (BMI, WC, HbA1c, HDL, LDL, TG, DBP, SBP, MAP, PR) and one MCS, the latter being derived from the core MetS components.

### 2.3 Genomic analyses

#### 2.3.1 Genotyping, quality control, and imputation

Genotype data for 2, 812 participants were generated on a customized Illumina IPMCN array with 570, 038 SNVs as described previously (49). Quality control (QC) and imputation were performed for 2, 812 GROUP participants with available genetic data. QC procedures were performed using PLINK v1.90 (50). SNVs and samples with missingness higher than 0.05 and 0.02, respectively, were removed. This led to strict and regular QC being conducted on 565, 901 SNVs and 2, 812 individuals.

**Figure 1:**
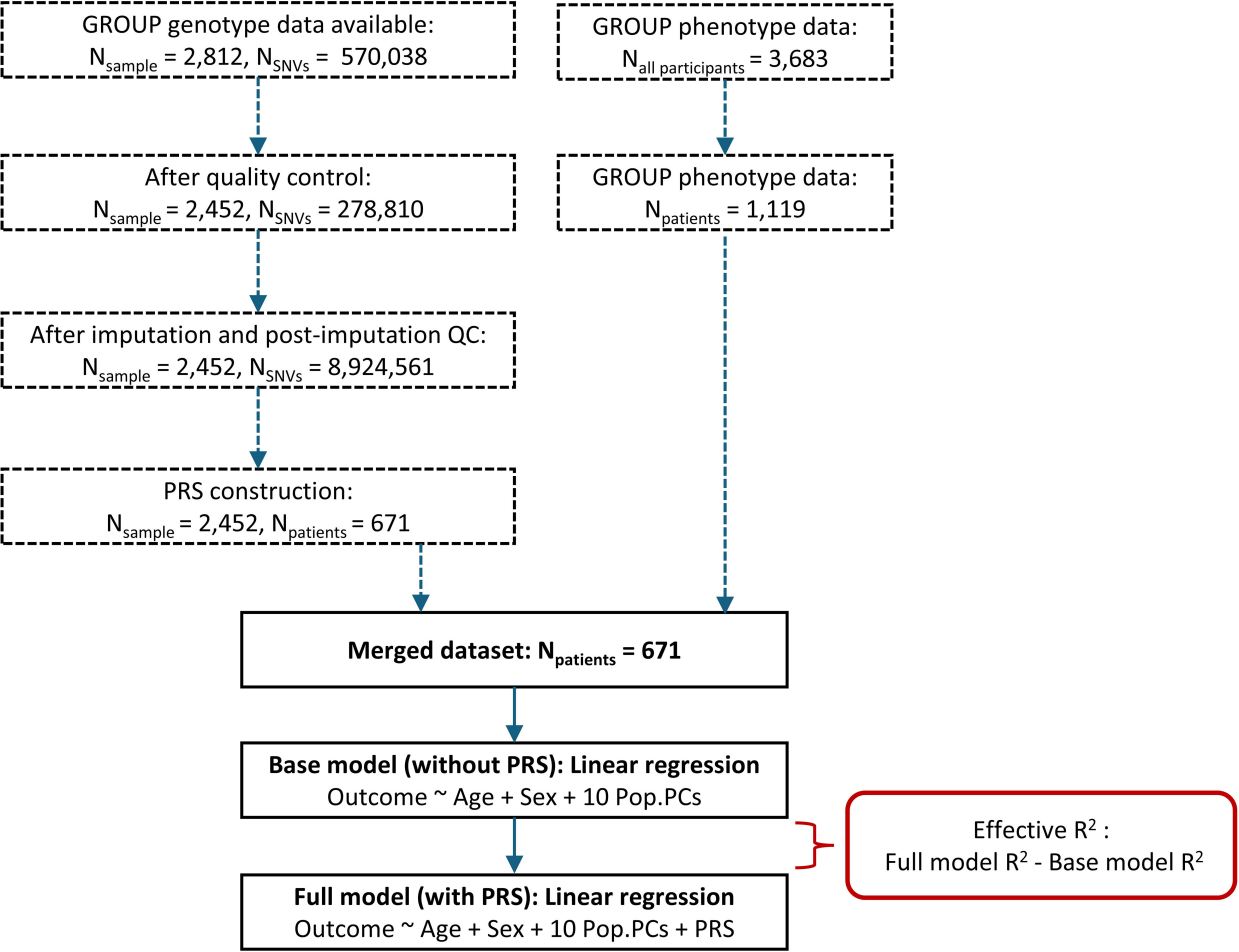
Flowchart of sample selection and data analysis.

A stringent QC process was implemented for SNVs, involving a minor allele frequency (MAF) threshold > 10%, Hardy-Weinberg Equilibrium (HWE) *P*-value> 1×10^-5^, and linkage disequilibrium (LD) r^2^ < 0.2, where r^2^ is the squared correlation between allelic values at two loci. This process resulted in the retention of 79, 870 high-quality SNVs. Subsequently, a thorough sample QC was performed to identify and exclude problematic samples. Samples were filtered based on sex discrepancy, heterozygosity (if the inbreeding coefficient F deviated from the mean by more than ±3 SD), and the identification of duplicate samples (Pihat > 0.8). A total of 167 samples were removed during these strict QC steps. An additional standard, less strict SNV QC was carried out with the criteria of SNV missingness < 2%, HWE *P*-value > 1×10^-06^, and MAF > 1%. Population stratification was conducted using multi-dimensional scaling (MDS) by clustering with individuals from 1000 Genome (1KG) Phase 1 (51). Outliers that deviated more than 3SD of European ancestry of principal component (PC) 1 and PC2 from 1KG individuals were removed (n=184, **Supplementary Figure 1**). Subsequent QC steps involved the removal of strand ambiguous and duplicate SNVs. Mendelian errors and missingness checks (0.02 for samples and SNVs) were followed. A final dataset comprising 2, 452 individuals and 278, 810 SNVs met all QC criteria (**Figure 1**).

Non-genotyped SNVs were imputed using the Trans-Omics for Precision Medicine (TOPMed) server (2) employing the TOPMed r2 reference panel for a mixed population. Before imputation, phasing was performed using Eagle v2.4. Post-imputation QC was conducted to ensure the reliability of the imputed SNVs. SNVs with an imputation quality score (r^2^) < 0.3 and MAF <1% were removed. Finally, 8, 924, 561 SNVs and 2, 452 individuals were retained for following genetic analyses. After merging with phenotype data, 671 patients with SSDs were included in the study.

#### 2.3.2 PRS construction for CRP and IL-6

Summary statistics for CRP (37) and IL-6 (38) obtained from our recently published meta-GWAS results of European individuals, ensuring their independence from GROUP participants. Clumping and thresholding (C+T) method was used to construct PRS_CRP_ and PRS_IL-6_ by PRSice2 (52). A range of *P*-value thresholds of <5x10^−8^, <5x10^−6^, <0.05, < 0.1, <0.2, <0.5, and <1.0 were tested with distance thresholds of 250kb and LD r^2^ thresholds of 0.1. R package *rtracklayer* (53) was used to lift over the GROUP genetic data with summary statistics.

### 2.4 Data analyses

The distribution of cardiometabolic outcomes was assessed using quantile-quantile plot before model fit. PRSes were standardized by subtracting the sample mean from an individual score and dividing the difference by sample SD. Linear regression models were fitted using standardized PRS_CRP_ and PRS_IL-6_ as independent variables, and cardiometabolic outcomes (BMI, WC, HbA1c, HDL, LDL, TG, SBP, DBP, MAP, PR, MCS) as dependent variables. The models were adjusted for age, sex, and the first 10 PCs of genetic ancestry. For each cardiometabolic, two linear regression models were fitted. Model I focused on the association of covariables (age, sex, PCs) and cardiometabolic outcomes, while model II incorporated the PRS, covariables, and cardiometabolic outcomes. We used the unadjusted R squared (R^2^) derived by subtracting the R^2^ of model I from that of model II to assess the variance explained specifically by PRSes (**Figure 1**). A bootstrapping method with replacement (54) was performed with 1, 000 resamples, to evaluate the model’s stability and reliability using the *boot* package in R. Specifically, for each resampled dataset, the linear regression model was refitted using the same sample size as the original dataset, allowing individual observations to appear multiple times. The distribution of the bootstrapped coefficients was then utilized to calculate confidence intervals and assess the robustness of the results. Besides, multiple testing was conducted using false discovery rate method to correct for false-positive estimates. All statistical analyses were conducted using the R (version 4.4.1) programming language (55).

### 2.5 Sensitivity analyses

Other potential modifiers including smoking and the use of antipsychotics showed non-significant and negligible associations with cardiometabolic outcomes in our previous study (6), and were therefore excluded from adjustment in the primary analyses. However, to evaluate the robustness of the observed associations, we conducted a sensitivity analysis in which the primary regression models were further adjusted for cigarette use and antipsychotic medication use. The number of cigarettes smoked per day was included as a continuous covariate, whereas antipsychotic medication use was included as a categorical covariate classified according to the risk of metabolic side effects. The detailed classification approach has been described previously (6). The same modeling strategy, bootstrapping procedure, and multiple-testing correction described above were applied in the sensitivity analyses.

## 3 Results

### 3.1 Baseline characteristics

The mean (± SD) age of the patients included in our study was 30.32 (± 7.02) years, ranging from 18 to 54 years, with the majority of them were males (74.81%). Patients had a mean (± SD) illness duration of 8.18 (± 3.97) years and 30.1% of them had experienced at least one psychotic episode since enrollment. Approximately 23.55% of the included patients met the criteria for MetS. The mean (± SD) values of MetS components were 34.91 (± 6.09) mmol/mol of HbA1c, 94.82 (± 13.86) cm of WC, 1.82 (± 1.24) mmol/mol of TG, 1.27 (± 0.75) mmol/mol of HDL, 79.72 (± 11.53) mmHg of DBP, and 127.54 (± 15.75) mmHg of SBP. Detailed characteristics of other cardiometabolic outcomes are presented in **Table 1**.

**Table 1:** Characteristics of included participants (N = 671).

Demographic and sociological characteristics	
Age, mean (SD)	30.32 (7.02)
Sex, male, n (%)	502 (74.81)
Years of education, mean (SD)	12.37 (3.79)
Marital status, n (%)	
Not married	554 (82.56)
Married/Living together	77 (11.48)
Other (divorced and widowed)	18 (2.68)
Estimated IQ, mean (SD)	100.18 (16.82)
Age onset illness, mean (SD)	23.39 (8.42)
Duration of illness in year, mean (SD)	8.18 (3.97)
Number of psychotic episodes since baseline, n (%)	
At least 1 episode	202 (30.10)
No episode	317 (47.24)
Status of antipsychotics, n (%)	
Not currently using	19(2.83)
Currently using	358(53.35)
Unknown if currently using	82(12.22)
Missing	212(31.59)
Cardiometabolic profiles	
BMI (kg/m^2^), mean (SD)	25.88 (4.54)
WC (cm), mean (SD)	94.82 (13.86)
HbA1c (mmol/mol), mean (SD)	34.91 (6.09)
HDL (mmol/l), mean (SD)	1.27 (0.75)
LDL (mmol/l), mean (SD)	3.10 (0.93)
TG (mmol/l), mean (SD)	1.82 (1.24)
DBP (mmHg), mean (SD)	79.72 (11.53)
SBP (mmHg), mean (SD)	127.54 (15.75)
MAP (mmHg), mean (SD)	95.66(11.66)
PR (beat/min), mean (SD)	74.97 (15.68)
MetS, n (%)	158 (23.55)

BMI, body mass index (kg/m^2^); WC, waist circumference (cm); HbA1c, glycated hemoglobin (mmol/mol); HDL, high density lipoprotein cholesterol (mmol/l); LDL, low density lipoprotein cholesterol (mmol/l); TG, triglycerides (mmol/l); SBP, systolic blood pressure (mmHg); DBP, diastolic blood pressure (mmHg); MAP, mean arterial pressure (mmHg); PR, pulse rate (beats/min); MetS, metabolic syndrome; SD, standard deviation.

### 3.2 Association between PRS_CRP_, PRS_IL-6_ and cardiometabolic outcomes

**Figure 2:**
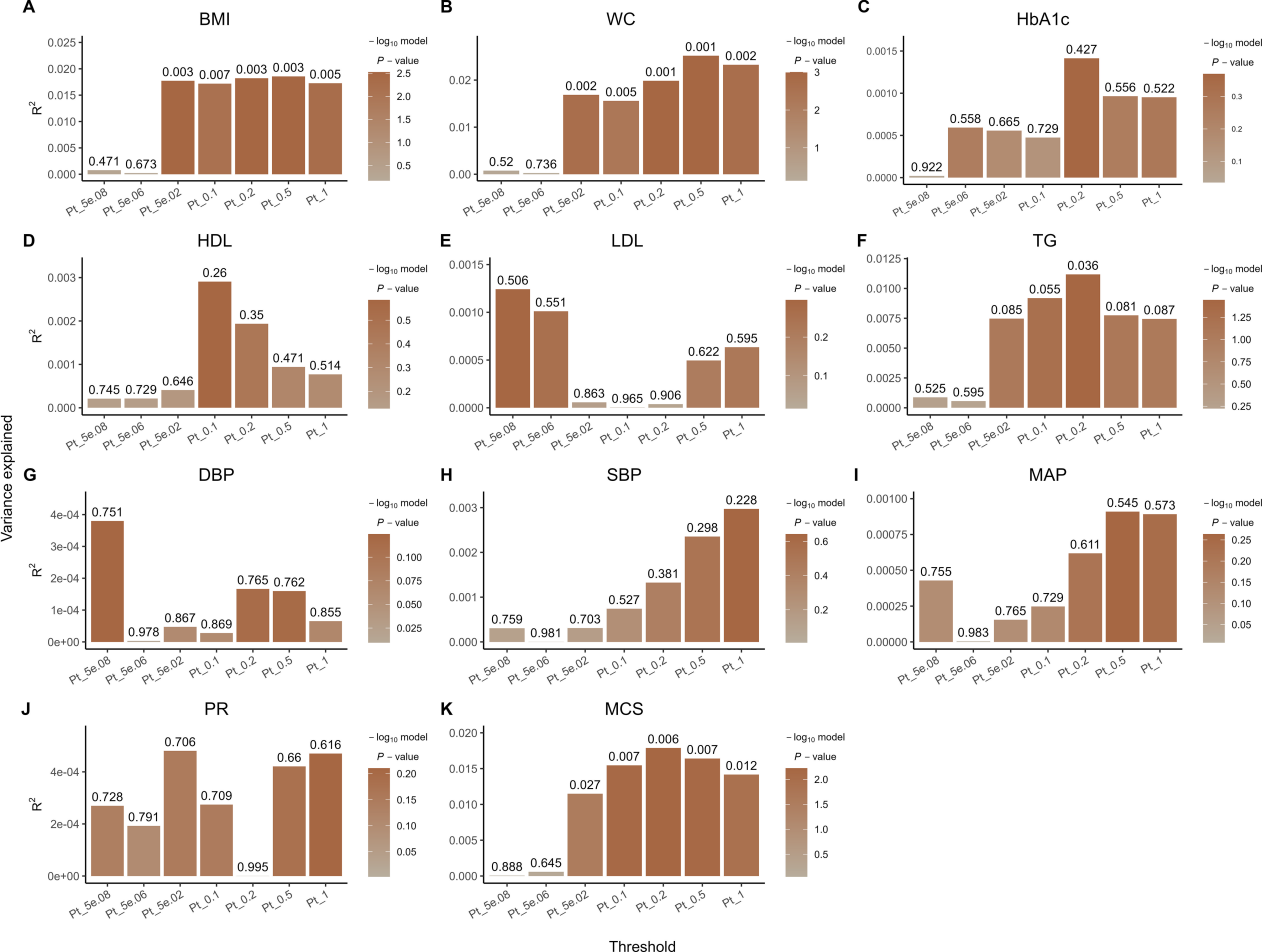
Explained variation of cardiometabolic outcomes by PRS_CRP_ CRP, C-reactive protein; BMI, body mass index (kg/m^2^); WC, waist circumference (cm); HbA1c, glycated hemoglobin (mmol/mol); HDL, high density lipoprotein cholesterol (mmol/l); LDL, low density lipoprotein cholesterol (mmol/l); TG, triglycerides (mmol/l); SBP, systolic blood pressure (mmHg); DBP, diastolic blood pressure (mmHg); MAP, mean arterial pressure (mmHg); PR, pulse rate (beats/min); MCS, metabolic composite score. *P* value threshold levels: Pt_5e_08: *P* value = 5x10^−8^; Pt_5e_06: *P* value = 5x10^−6^; Pt_5e_02: *P* value =0.05; Pt_0.1: *P* value =0.1; Pt_0.2: *P* value =0.2; Pt_0.5: *P* value =0.5; Pt_1: *P* value =1. **(A)** Explained variation of BMI by PRSCRP. **(B)** Explained variation of WC by PRSCRP. **(C)** Explained variation of HbA1c by PRSCRP. **(D)** Explained variation of HDL by PRSCRP. **(E)** Explained variation of LDL by PRSCRP. **(F)** Explained variation of TG by PRSCRP. **(G)** Explained variation of DBP by PRSCRP. **(H)** Explained variation of SBP by PRSCRP. **(I)** Explained variation of MAP by PRSCRP. **(J)** Explained variation of PR by PRSCRP. **(K)** Explained variation of MCS by PRSCRP.

Standardized PRS_CRP_ was significantly associated with increased BMI (β_Pt_0.5_ = 0.64, 95%CI=0.21 to 1.02, *P_bootstapping_* = 0.003), WC (β_Pt_0.5_ = 2.25, 1.00 to 3.53, *P_bootstapping_* < 0.001), TG levels (β_Pt_0.2_ = 0.13, 0.01 to 0.26, *P_bootstapping_* = 0.036), and MCS (β_Pt_0.2_ =0.14, 0.04 to 0.24, *P_bootstapping_* = 0.006) (**Table 2**). Among those with significant associations, PRS_CRP_ at *P-*value threshold of 0.5 explained the most variance in BMI (1.85%) and WC (2.52%). PRS_CRP_ at a *P*-value threshold of 0.2 that included 160, 994 SNVs jointly explained the most variance in TG (1.12%) and MCS (1.79%) (**Figure 2**). However, the association between PRS_CRP_ and TG levels, as well as MCS disappeared after correcting for multiple testing.

**Table 2:** Association of PRS_CRP_ and PRS_IL-6_ with cardiometabolic outcomes (N = 671).

Predictor β (SE)	Outcome										
	BMI	WC	HbA1c	HDL	LDL	TG	SBP	DBP	MAP	PR	MCS
PRS_CRP_											
PRS_Pt_5e08_	-0.16 (0.22)	-0.45 (0.70)	-0.03 (0.28)	0.01 (0.03)	-0.03 (0.05)	0.04 (0.07)	-0.26 (0.85)	-0.20 (0.62)	-0.20 (0.64)	-0.27 (0.79)	0.01 (0.05)
PRS_Pt_5e06_	-0.09 (0.22)	-0.23 (0.69)	0.16 (0.27)	0.01 (0.03)	-0.03 (0.05)	0.04 (0.07)	-0.02 (0.78)	0.02 (0.60)	-0.01 (0.64)	-0.21 (0.79)	0.03 (0.05)
PRS_Pt_0.05_	0.64 (0.22)^**^	1.87 (0.60)^**^	0.15 (0.33)	-0.02 (0.04)	-0.01 (0.05)	0.11 (0.07)	0.28 (0.74)	0.09 (0.53)	0.16 (0.54)	0.31 (0.83)	0.12 (0.05) †
PRS_Pt_0.1_	0.61 (0.22)^**^	1.81 (0.64)^**^	0.14 (0.33)	-0.04 (0.04)	0.00 (0.05)	0.12 (0.06)	0.49 (0.77)	0.09 (0.58)	0.19 (0.55)	0.29 (0.78)	0.14 (0.05) †
PRS_Pt_0.2_	0.63 (0.21)^**^	2.01 (0.61)^**^	0.23 (0.29)	-0.03 (0.04)	0.01 (0.05)	0.13 (0.06) †	0.61 (0.69)	0.16 (0.53)	0.28 (0.56)	-0.00 (0.74)	0.14 (0.05) †
PRS_Pt_0.5_	0.64 (0.21)^**^	2.25 (0.65)^**^	0.18 (0.30)	-0.02 (0.03)	-0.02 (0.05)	0.11 (0.06)	0.78 (0.75)	0.18 (0.58)	0.35 (0.58)	-0.33 (0.76)	0.14 (0.05) †
PRS_Pt_1_	0.62 (0.22)^**^	2.16 (0.69)^**^	0.20 (0.31)	-0.02 (0.03)	-0.03 (0.05)	0.11 (0.07)	0.92 (0.76)	0.11 (0.58)	0.33 (0.58)	-0.39 (0.77)	0.13 (0.05) †
PRS_IL-6_											
PRS_Pt_5e08_	-0.05 (0.20)	0.36 (0.62)	-0.48 (0.23) †	-0.04 (0.03)	0.02 (0.05)	-0.03 (0.06)	-0.01 (0.72)	-0.57 (0.48)	-0.38 (0.52)	1.21 (0.79)	-0.04 (0.05)
PRS_Pt_5e06_	-0.12 (0.20)	0.10 (0.61)	-0.65 (0.25) †	-0.03 (0.03)	-0.01 (0.05)	-0.03 (0.06)	-0.18 (0.72)	-0.86 (0.49)	-0.65 (0.53)	1.10 (0.79)	-0.07 (0.05)
PRS_Pt_0.05_	-0.24 (0.21)	-0.62 (0.65)	-0.30 (0.31)	0.00 (0.04)	0.04 (0.05)	0.06 (0.06)	-0.97 (0.79)	-1.07 (0.55) †	-1.04 (0.55)	-0.15 (0.68)	-0.05 (0.06)
PRS_Pt_0.1_	-0.14 (0.22)	-0.13 (0.62)	-0.25 (0.32)	0.00 (0.04)	0.02 (0.05)	0.10 (0.06)	-0.36 (0.75)	-0.48 (0.51)	-0.42 (0.56)	-0.11 (0.70)	0.01 (0.06)
PRS_Pt_0.2_	-0.14 (0.22)	-0.06 (0.64)	-0.54 (0.36)	0.00 (0.05)	0.02 (0.05)	0.09 (0.07)	-0.71 (0.82)	-0.46 (0.59)	-0.53 (0.57)	-0.08 (0.76)	0.02 (0.06)
PRS_Pt_0.5_	-0.10 (0.21)	-0.02 (0.63)	-0.49 (0.42)	0.00 (0.04)	-0.03 (0.05)	0.08 (0.06)	-0.38 (0.84)	-0.50 (0.62)	-0.45 (0.60)	-0.12 (0.70)	0.01 (0.06)
PRS_Pt_1_	-0.13 (0.23)	-0.31 (0.66)	-0.44 (0.41)	0.01 (0.04)	-0.03 (0.05)	0.07 (0.06)	-0.29 (0.83)	-0.49 (0.61)	-0.40 (0.62)	-0.05 (0.76)	-0.00 (0.06)

Each model was adjusted by age, sex, and 10 PCs of population structure.

The coefficients provided were estimated using bootstrapping with 1, 000 resamples.

Significance level: ^†^Nominally significant at raw *P* < 0.05; **FDR-adjusted *P* < 0.05

*P* value threshold levels: Pt_5e_08: *P* value = 5x10^−8^; Pt_5e_06: *P* value = 5x10^−6^; Pt_5e_02: *P* value =0.05; Pt_0.1: *P* value =0.1; Pt_0.2: *P* value =0.2; Pt_0.5: *P* value =0.5; Pt_1: *P* value =1.

CRP, C-reactive protein; BMI, body mass index (kg/m^2^); WC, waist circumference (cm); HbA1c, glycated hemoglobin (mmol/mol); HDL, high density lipoprotein cholesterol (mmol/l); LDL, low density lipoprotein cholesterol (mmol/l); TG, triglycerides (mmol/l); SBP, systolic blood pressure (mmHg); DBP, diastolic blood pressure (mmHg); MAP, mean arterial pressure (mmHg); PR, pulse rate (beats/min); MCS, metabolic composite score; SE, standard error.

**Figure 3:**
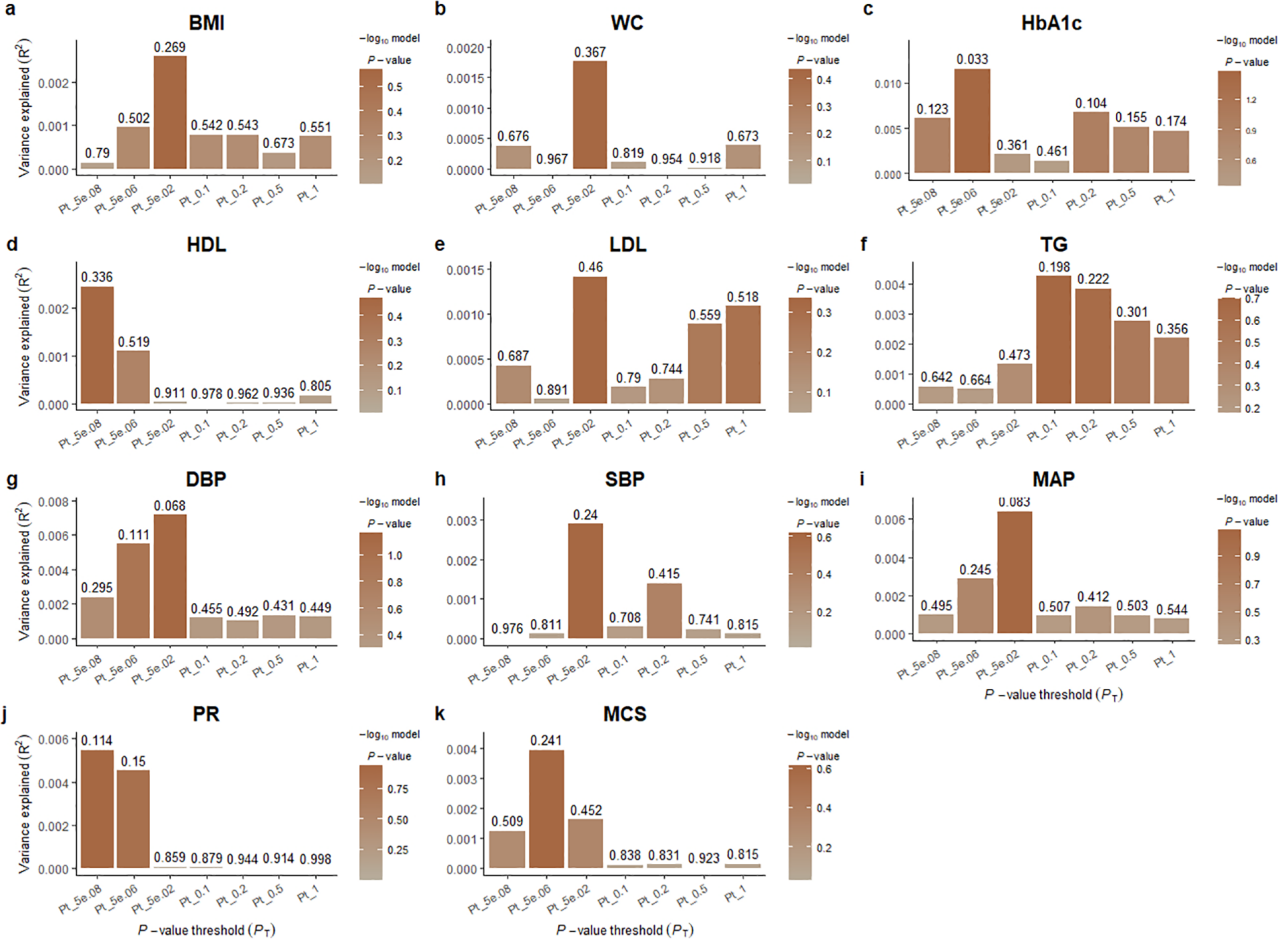
Explained variation of cardiometabolic outcomes by PRS_IL-6._ IL-6, Interleukin-6; BMI, body mass index (kg/m^2^); WC, waist circumference (cm); HbA1c, glycated hemoglobin (mmol/mol); HDL, high density lipoprotein cholesterol (mmol/l); LDL, low density lipoprotein cholesterol (mmol/l); TG, triglycerides (mmol/l); SBP, systolic blood pressure (mmHg); DBP, diastolic blood pressure (mmHg); MAP, mean arterial pressure (mmHg); PR, pulse rate (beats/min); MCS, metabolic composite score. *P* value threshold levels: Pt_5e_08: *P* value = 5x10^−8^; Pt_5e_06: *P* value = 5x10^−6^; Pt_5e_02: *P* value =0.05; Pt_0.1: *P* value =0.1; Pt_0.2: *P* value =0.2; Pt_0.5: *P* value =0.5; Pt_1: *P* value =1. **(a)** Explained variation of BMI by PRSIL-6. **(b)** Explained variation of WC by PRSIL-6. **(c)** Explained variation of HbA1c by PRSIL-6. **(d)** Explained variation of HDL by PRSIL-6. **(e)** Explained variation of LDL by PRSIL-6. **(f)** Explained variation of TG by PRSIL-6. **(g)** Explained variation of DBP by PRSIL-6. **(h)** Explained variation of SBP by PRSIL-6. **(i)** Explained variation of MAP by PRSIL-6. **(j)** Explained variation of PR by PRSIL-6. **(k)** Explained variation of MCS by PRSIL-6.

We found that the increased standardized PRS_IL-6_ was associated with decreased HbA1c level (β_Pt_5e06_=-0.66, -1.26 to -0.05, *P_bootstapping_* = 0.033) at a *P*-value threshold of 5x10^−6^ and explained 1.16% of the variance (**Figure 3**). This association did not remain significant after adjusting for multiple testing.

### 3.3 Sensitivity analyses results

Sensitivity analyses additionally adjusting for cigarette use and antipsychotic medication use yielded results largely consistent with those of the primary analyses (**Supplementary Table 1**). The associations between PRS_CRP_ and obesity-related traits remained robust, with FDR-significant associations observed for BMI and WC across the same PRS thresholds as in the primary analyses. Effect estimates were highly comparable before and after adjustment. In addition, associations between PRS_CRP_ and MCS were modestly strengthened after adjustment, with several thresholds reaching FDR significance. In contrast, PRS_IL-6_ continued to show no consistent associations with cardiometabolic outcomes.

## 4 Discussion

We have investigated the associations between PRSes for inflammatory biomarkers and cardiometabolic outcomes in patients with SSD in the Netherlands. We found that PRS_CRP_, but not PRS_IL-6_ was associated with increased BMI, WC, TG and MCS, with the phenotypic variance explained ranging between 1.12% for TG to 2.52% for WC.

### 4.1 PRS_CRP_ and cardiometabolic outcomes

Genetic predisposition to low-grade inflammation, indicated by PRS_CRP_, correlates with increased obesity markers such as BMI, waist circumference, and triglycerides, but not with LDL or HDL cholesterol levels. The research suggests this genetic factor has a modest, additive effect on obesity-related outcomes in individuals with SSDs over three years. Additionally, PRS_CRP_ is significantly associated with heightened risk of metabolic disturbances, as reflected by MCS scores, highlighting its role in metabolic health evaluation.

Phenotypically, MetS with its components have consistently been correlated with CRP levels across diverse population (56–61). Likewise, abdominal obesity is a common comorbidity in patients with SSDs (62) and closely related to CRP (63). BMI and WC were moderately correlated with CRP ranging from an index of 0.31 to 0.40 (64). However, genetic associations showed inconsistency across studies (65, 66). Part of the research variability could be attributable by the difference between different ethinic groups. For instance, a functional CRP SNV rs3091244 was associated with MetS in Chinese (67), but not with Iranian population (68).

Here we also face a differential association between general and patient populations. Our previous large-scale mendelian randomization study demonstrated significant causal effects of PRS_CRP_ on elevated blood pressures but not on BMI in the general population (69, 70). In contrast, another study (40) demonstrated that CRP SNVs are significantly associated with HDL, LDL, TG, and BMI levels using summary statistics from large population-based biobanks. Similarly, although individual CRP SNV have been shown an additive effect on fasting insulin and the risk of T2DM (71, 72) in the general populations, we did not observe such an association in patients with SSDs 3 years after diagnosis. We speculate that disease status and medical interventions mask the genetic effects of PRS_CRP_ on these otherwise susceptible outcomes. This question calls for further studies and will illustrate whether genetic risk is implicated in blood related outcomes in SSDs.

Our findings should also be interpreted within the broader context of evidence indicating that SSD and other psychiatric disorders are characterized by a chronic low-grade inflammatory state. Previous studies have consistently reported elevated circulating levels of inflammatory biomarkers such as CRP, IL-6, and TNF-α in individuals with SSDs (19, 73). Such inflammatory dysregulation has been implicated in both the symptom severity, cognitive dysfunction, and illness progression of psychosis and the development of cardiometabolic abnormalities. In this context, the observed associations between PRS_CRP_ and obesity-related traits suggest that genetic susceptibility to low-grade inflammation contributes to the increased cardiometabolic burden observed in SSDs. Individuals with a higher genetic predisposition to elevated CRP levels are subjected to persistent increased levels of inflammatory reactions, and thus to adipose tissue expansion, obesity and its consequential cardiometabolic complications (74). At the same time, the lack of significant associations with HbA1c or blood pressure should not be interpreted as evidence of no biological relationship, as these traits are influenced by multiple physiological and environmental factors and smaller effects may have gone undetected. Our findings support the notion that inflammation-related genetic factors may represent one pathway linking SSDs to adverse cardiometabolic outcomes. However, the relationships among low-grade inflammation, metabolic dysregulation, and severe mental illness including SSDs are complex and likely bidirectional (75). This creates a self-perpetuating cycle in which inflammation contributes to metabolic abnormalities, which in turn further promote inflammatory processes.

Antipsychotic medications, particularly second-generation antipsychotics, are well-known contributors to metabolic disturbances in patients with SSDs (76, 77). Therefore, antipsychotic treatment could potentially confound the association between inflammatory genetic susceptibility and cardiometabolic outcomes. However, our sensitivity analyses showed that the associations between PRS_CRP_ and obesity-related traits remained largely unchanged after adjusting for antipsychotic medication use. This findings suggests that the observed associations are unlikely to be explained by antipsychotic exposure and may reflect an independent contribution of genetic liability to low-grade inflammation. Nevertheless, as information on cumulative dose, treatment duration, and detailed medication switching was not available in our study, residual confounding by antipsychotic treatment cannot be completely excluded. Future longitudinal studies incorporating detailed medication exposure data are warranted.

### 4.2 PRS_IL-6_ and cardiometabolic outcomes

While literature suggests IL6 mediating roles in glucose and lipid metabolism (78), we found no association between PRS_IL-6_ and most of the cardiometabolic outcome. Previously, IL-6 SNVs rs1800797 and rs1800796 were shown to be correlated with cardiometabolic outcomes in Mexian-Americans (79). SNVs in IL-6 genes have been shown to be associated with increased blood pressure in Japanese, and fasting insulin in Korean (71). In contrast, a recent study (80) found a protective effect on the risk for T2DM, but no effect on HbA1c and fasting glucose levels. Therefore, whether SNVs of IL-6 contributes to cardiometabolic outcomes in patients with SSDs remains to be determined. It should be noted that the sample size of the IL-6 GWAS source was smaller than the CRP discovery dataset. The fewer included SNVs in the PRS_IL-6_ construction might potentially impact our results due to reduced statistical power. In addition, the observed null associations might be attributable to fundamental biological differences between IL-6 and CRP. IL-6 is a pleiotropic cytokine (81) with both pro-inflammatory and anti-inflammatory properties (82) and participates in multiple immune, metabolic, and neuroendocrine pathways. Consequently, circulating IL-6 may reflect a heterogeneous range of biological processes (83). In contrast, CRP is a downstream acute-phase protein in response to inflammatory signaling (84), particularly IL-6, and is widely regarded as a stable marker of systemic low-grade inflammation. As obesity-related traits are closely linked to persistent inflammatory activity, genetic predisposition to elevated CRP levels may capture cumulative inflammatory burden and cardiometabolic risk more effectively than genetic predisposition to IL-6 levels alone. This may partly explain why PRS_CRP_ showed more robust associations with obesity-related traits than PRS_IL-6_ in the present study.

### 4.3 Clinical implication and study limitations

Although the need for screening and monitoring of MetS and its components has been acknowledged in psychiatric treatment guidelines (85, 86), the testing for metabolic risk factors particularly glucose, was much less frequent among patients with SSDs than those with T2DM (87). Understanding the genetic components underlying cardiometabolic risk in SSDs may help identify individuals at higher risk, particularly among those treated with psychotropic medications. For example, PRS_CRP_ could potentially inform clinical decision-making by identifying individuals with greater genetic liability to low-grade inflammation, who may benefit from treatment strategies with lower metabolic risk. More broadly, inflammation-based stratification models may improve risk assessment and support targeted interventions for metabolic disorders (88).

This study has several limitations. First, although the GROUP study is a longitudinal cohort, the cardiometabolic outcomes examined in the present study were assessed at a single 3-year follow-up visit. Therefore, the current analyses are cross-sectional in nature and do not permit causal inference. Second, although we included commonly used covariates in the genetic analyses and conducted sensitivity analyses further adjusting for cigarette use and antipsychotic medication use, we were unable to account for other lifestyle and environmental factors such as diet, physical activity, or for gene–gene and gene–environment interactions that may modify genetic risk. Third, we did not account for rare‐variant effects in PRS estimations, and our scores were derived exclusively from European‐ancestry discovery datasets. Fourth, this study focused on PRSes for CRP and IL-6 and did not examine other inflammation-related biomarkers. Consequently, our findings may not fully capture the broader genetic susceptibility to inflammatory processes relevant to cardiometabolic dysfunction in SSDs. Future studies are recommended to investigate additional inflammatory pathways and biomarkers to provide a more comprehensive assessment of inflammation-related cardiometabolic risk. Furthermore, the study sample was predominantly male (74.8%) and relatively young (mean age approximately 30 years). Therefore, the findings may not be fully generalizable to female patients or older individuals with SSDs. As cardiometabolic abnormalities tend to accumulate with age, the magnitude and nature of the associations between inflammatory PRSs and cardiometabolic outcomes may differ in older populations.

## 5 Conclusions

We found that higher genetic susceptibility of low-grade inflammation measure by PRS for CRP was significantly associated with increased waist circumference and obesity-related lipid levels in patients with SSDs at 3 years after diagnosis independent from antipsychotics use. Our results offer a better understanding of genetic susceptibility to cardiometabolic outcomes and provide insights for an informed decision for antipsychotics choice for SSDs with high genetic risk for low-grade inflammation.

## Data Availability

The data analyzed in this study is subject to the following licenses/restrictions: Data from the GROUP cohort can be shared upon request. Researchers interested in accessing the data may contact the GROUP coordinator, LH (l.dehaan@amsterdamumc.nl), for information on application requirements. Shared data may only be used for the approved research proposal. Requests to access these datasets should be directed to LH, l.dehaan@amsterdamumc.nl.
