# Supplementary material for: C-reactive protein polygenic risk is associated with obesity-related traits in schizophrenia spectrum disorders
Source: Front Psychiatry. 2026 Jul 14;17:1873942. doi: 10.3389/fpsyt.2026.1873942 (PMC13407836; doi:10.3389/fpsyt.2026.1873942)
Supplement: Supplementary file 1 [file SupplementaryFile1.docx]

**C-reactive Protein Polygenic Risk is Associated with Obesity-related Traits in Schizophrenia Spectrum Disorders**

Chenxu Zhao et al.

Table of Contents

[Supplementary Methods 2](#_Toc232602540)

[Supplementary Figure 1. Multi-dimensional scaling plot of the top PCs, before (a) and after (b) removing European outliers. 3](#_Toc232602541)

[Supplementary Table S1. Sensitivity analysis results (N=671) 4](#_Toc232602542)

# **Supplementary Methods**

*Definition criteria for MetS*

MetS was defined according to the US National Cholesterol Education Programme Adult Treatment Panel III (NCEP-ATP-III) criteria, where the presence of any three of the following five features constituted a diagnosis of MetS(Grundy et al., 2005): i) fasting plasma glucose ≥100mg/dl (equivalent to 5.6mmol/l) or the use of antidiabetic medications(Forouhi et al., 2006). In cases where plasma glucose levels were not available, a HbA1c ≥5.1% (equivalent to 32mmol/mol) was used as a criterion(International Expert, 2009); ii) HDL cholesterol <50mg/dl (equivalent to 1.30mmol/l) in woman or <40mg/dl (equivalent to 1.03mmol/l) in men; or the use of HDL increasing drugs; iii) TG ≥150mg/dl (equivalent to 1.7mmol/l) or the use of triglyceride-lowering drugs; iv) BP ≥130/85mmHg or the use of antihypertensives; v) WC ≥88cm in women or 102cm in men.

Quality control, and imputation for genotype data

SNVs and samples with missingness higher than 0.05 and 0.02, respectively, were removed. This led to strict and regular QC being conducted on 565,901 SNVs and 2,812 individuals. A stringent QC process was implemented for SNVs, involving a minor allele frequency (MAF) threshold > 10%, Hardy-Weinberg Equilibrium (HWE) P-value > 1×10-5, and linkage disequilibrium (LD) r2<0.2. Subsequently, samples with sex discrepancy, high heterozygosity, duplicate samples were removed (n=167) during these strict QC steps. An additional standard, less strict SNV QC was carried out with the criteria of SNV missingness <2%, HWE P-value >1×10-06, and MAF >1%. Population stratification was conducted using multi-dimensional scaling (MDS) by clustering with individuals from 1000 Genome (1KG) Phase 1[45]. European outliers of principal component (PC)1 and PC2 from 1KG individuals were removed (n=184, Supplementary Figure S1). Strand ambiguous and duplicate SNVs, mendelian errors, and missingness checks (0.02 for samples and SNVs) were followed. Before imputation, phasing was performed using Eagle v2.4. Post-imputation QC was conducted by removing SNVs with an imputation quality score (INFO) < 0.3 and MAF < 1%.


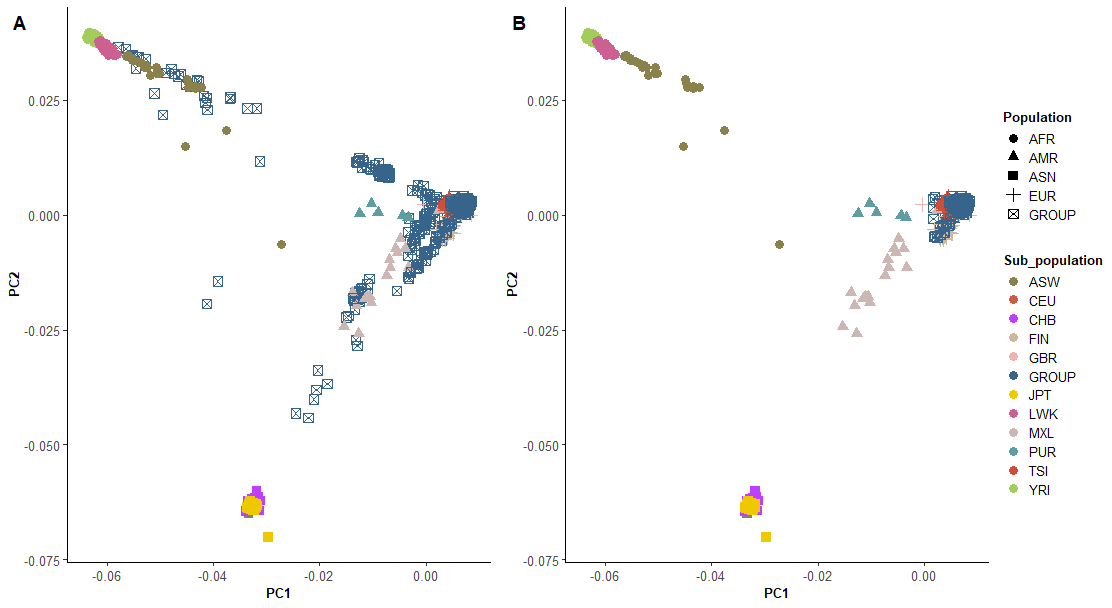


# **Supplementary Figure 1. Multi-dimensional scaling plot of the top PCs, before (a) and after (b) removing European outliers.**

Abbreviations: PC: principal component; AFR: African ancestry; AMR: American ancestry; ASN: Asian ancestry; EUR: European ancestry; GROUP: GROUP participants; ASW: African Ancestry SW; CEU: CEPH; CHB: Han Chinese; FIN: Finish; GBR: British; JPT: Japanese; LWK: luhya; MXL: Mexican Ancestry; PUR: Puerto Rican; TSI: Toscani; YRI: Yoruba

# **Supplementary Table S1. Sensitivity analysis results (N=671)**

| **Predictor**  **β (SE)** | **Outcome** | | | | | | | | | | |
| --- | --- | --- | --- | --- | --- | --- | --- | --- | --- | --- | --- |
|  | **BMI** | **WC** | **HbA1c** | **HDL** | **LDL** | **TG** | **SBP** | **DBP** | **MAP** | **PR** | **MCS** |
| **PRS_CRP_** |  |  |  |  |  |  |  |  |  |  |  |
| PRS_Pt_5e08_ | -0.12(0.24) | -0.42(0.80) | 0.14(0.31) | 0.01(0.04) | -0.03(0.06) | 0.01(0.07) | 0.02(0.96) | -0.15(0.68) | -0.13(0.72) | -0.47(0.89) | 0.01(0.06) |
| PRS_Pt_5e06_ | 0.00(0.25) | -0.15(0.76) | 0.24(0.29) | 0.01(0.04) | -0.04(0.06) | 0.01(0.07) | 0.17(0.90) | 0.22(0.68) | 0.18(0.69) | -0.40(0.94) | 0.03(0.06) |
| PRS_Pt_0.05_ | 0.66(0.22)^**^ | 1.92(0.65)^**^ | 0.38(0.37) | -0.03(0.04) | -0.00(0.05) | 0.11(0.07) | 0.04(0.81) | 0.12(0.58) | 0.06(0.61) | 0.49(0.94) | 0.14(0.06)^†^ |
| PRS_Pt_0.1_ | 0.63(0.23)^**^ | 1.88(0.69)^**^ | 0.45(0.35) | -0.06(0.04) | 0.01(0.05) | 0.12(0.07) | 0.15(0.85) | 0.19(0.62) | 0.13(0.61) | 0.55(0.91) | 0.18(0.06)^**^ |
| PRS_Pt_0.2_ | 0.70(0.23)^**^ | 2.06(0.67)^**^ | 0.45(0.34) | -0.05(0.04) | 0.02(0.05) | 0.13(0.07) | 0.35(0.82) | 0.34(0.61) | 0.36(0.61) | 0.13(0.88) | 0.17(0.06)^**^ |
| PRS_Pt_0.5_ | 0.67(0.23)^**^ | 2.32(0.69)^**^ | 0.37(0.36) | -0.04(0.04) | -0.00(0.05) | 0.11(0.07) | 0.45(0.80) | 0.31(0.65) | 0.37(0.66) | -0.02(0.87) | 0.17(0.06)^**^ |
| PRS_Pt_1_ | 0.65(0.23)^**^ | 2.16(0.72)^**^ | 0.36(0.34) | -0.03(0.04) | -0.01(0.05) | 0.11(0.07) | 0.56(0.92) | 0.29(0.65) | 0.37(0.63) | -0.04(0.87) | 0.16(0.06)^**^ |
| **PRS_IL-6_** |  |  |  |  |  |  |  |  |  |  |  |
| PRS_Pt_5e08_ | -0.03(0.22) | 0.05(0.68) | -0.45(0.28) | -0.05(0.03) | 0.04(0.05) | -0.06(0.07) | 0.08(0.78) | -0.56(0.57) | -0.36(0.59) | 2.32(0.86)^†^ | -0.06(0.06) |
| PRS_Pt_5e06_ | -0.03(0.23) | -0.01(0.72) | -0.52(0.30) | -0.03(0.04) | 0.03(0.05) | -0.05(0.08) | -0.05(0.81) | -0.79(0.58) | -0.58(0.58) | 2.32(0.81)^†^ | -0.08(0.06) |
| PRS_Pt_0.05_ | -0.26(0.23) | -0.46(0.70) | -0.12(0.24) | -0.01(0.05) | 0.07(0.05) | 0.08(0.07) | -1.60(0.81)^†^ | -0.83(0.57) | -1.11(0.60) | 0.42(0.77) | 0.02(0.07) |
| PRS_Pt_0.1_ | -0.19(0.25) | 0.05(0.70) | -0.01(0.25) | -0.03(0.05) | 0.05(0.05) | 0.13(0.08) | -1.02(0.76) | -0.39(0.58) | -0.60(0.58) | 0.42(0.81) | 0.08(0.06) |
| PRS_Pt_0.2_ | -0.13(0.24) | 0.32(0.70) | -0.23(0.28) | -0.01(0.05) | 0.05(0.06) | 0.14(0.08) | -1.16(0.91) | -0.30(0.65) | -0.56(0.64) | 0.67(0.85) | 0.08(0.07) |
| PRS_Pt_0.5_ | -0.06(0.25) | 0.30(0.77) | -0.22(0.34) | -0.02(0.05) | -0.00(0.06) | 0.12(0.07) | -0.57(0.91) | -0.08(0.62) | -0.26(0.69) | 0.74(0.83) | 0.07(0.07) |
| PRS_Pt_1_ | -0.10(0.26) | 0.04(0.76) | -0.19(0.31) | 0.00(0.05) | -0.01(0.06) | 0.11(0.07) | -0.39(0.97) | -0.03(0.63) | -0.15(0.66) | 0.93(0.82) | 0.05(0.07) |
| Each model was adjusted by age, sex, top 10 PCs of population structure., cigarette use per day, and use of antipsychotics.  The coefficients provided were estimated using bootstrapping with 1,000 resamples.  Significance level: ^†^ Nominally significant at raw *P* < 0.05; ^**^ FDR-adjusted *P* < 0.05  *P* value threshold levels: Pt_5e_08: *P* value = 5x10^−8^; Pt_5e_06: *P* value = 5x10^−6^; Pt_5e_02: *P* value =0.05; Pt_0.1: *P* value =0.1; Pt_0.2: *P* value =0.2; Pt_0.5: *P* value =0.5; Pt_1: *P* value =1.  Abbreviations: CRP**:** C-reactive protein; BMI: body mass index (kg/m^2^); WC: waist circumference (cm); HbA1c: glycated hemoglobin (mmol/mol); HDL: high density lipoprotein cholesterol (mmol/l); LDL: low density lipoprotein cholesterol (mmol/l); TG: triglycerides (mmol/l); SBP: systolic blood pressure (mmHg); DBP: diastolic blood pressure (mmHg); MAP: mean arterial pressure (mmHg); PR: pulse rate (beats/min); MCS: metabolic composite score; SE: standard error. | | | | | | | | | | | |

**References**

Forouhi, N. G., Balkau, B., Borch-Johnsen, K., Dekker, J., Glumer, C., Qiao, Q., Spijkerman, A., Stolk, R., Tabac, A., Wareham, N. J. and Edeg (2006) 'The threshold for diagnosing impaired fasting glucose: a position statement by the European Diabetes Epidemiology Group', *Diabetologia,* 49(5), pp. 822-7. DOI: 10.1007/s00125-006-0189-4.

Grundy, S. M., Cleeman, J. I., Daniels, S. R., Donato, K. A., Eckel, R. H., Franklin, B. A., Gordon, D. J., Krauss, R. M., Savage, P. J. and Smith Jr, S. C. (2005) 'Diagnosis and management of the metabolic syndrome: an American Heart Association/National Heart, Lung, and Blood Institute scientific statement', *Circulation,* 112(17), pp. 2735-2752. DOI: 10.1097/01.hco.0000200416.65370.a0. .

International Expert, C. (2009) 'International Expert Committee report on the role of the A1C assay in the diagnosis of diabetes', *Diabetes Care,* 32(7), pp. 1327-34. DOI: 10.2337/dc09-9033.
